# HAMdb: a database of human autophagy modulators with specific pathway and disease information

**DOI:** 10.1186/s13321-018-0289-4

**Published:** 2018-07-31

**Authors:** Ning-Ning Wang, Jie Dong, Lin Zhang, Defang Ouyang, Yan Cheng, Alex F. Chen, Ai-Ping Lu, Dong-Sheng Cao

**Affiliations:** 1 Xiangya School of Pharmaceutical Sciences, Central South University, No. 172, Tongzipo Road, Yuelu District, Changsha, People’s Republic of China; 2 Hunan Key Laboratory of Grain-Oil Deep Process and Quality Control, Hunan Key Laboratory of Processed Food for Special Medical Purpose, National Engineering Laboratory for Deep Processing of Rice and Byproducts, Central South University of Forestry and Technology, Changsha, People’s Republic of China; 3 State Key Laboratory of Quality Research in Chinese Medicine, Institute of Chinese Medical Sciences (ICMS), University of Macau, Macau, People’s Republic of China; 4 Center for Vascular Disease and Translational Medicine, The Third Xiangya Hospital of Central South University, Changsha, People’s Republic of China; 5 Institute for Advancing Translational Medicine in Bone and Joint Diseases, School of Chinese Medicine, Hong Kong Baptist University, Hong Kong, SAR, People’s Republic of China

**Keywords:** Autophagy, Autophagy modulator, Database, Disease, Pathophysiological, Pathway

## Abstract

Autophagy is an important homeostatic cellular recycling mechanism responsible for degrading unnecessary or dysfunctional cellular organelles and proteins in all living cells. In addition to its vital homeostatic role, this degradation pathway also involves in various human disorders, including metabolic conditions, neurodegenerative diseases, cancers and infectious diseases. Therefore, the comprehensive understanding of autophagy process, autophagy-related modulators and corresponding pathway and disease information will be of great help for identifying the new autophagy modulators, potential drug candidates, new diagnostic and therapeutic targets. In recent years, some autophagy databases providing structural and functional information were developed, but the specific databases covering autophagy modulator (proteins, chemicals and microRNAs)-related target, pathway and disease information do not exist. Hence, we developed an online resource, Human Autophagy Modulator Database (HAMdb, http://hamdb.scbdd.com), to provide researchers related pathway and disease information as many as possible. HAMdb contains 796 proteins, 841 chemicals and 132 microRNAs. Their specific effects on autophagy, physicochemical information, biological information and disease information were manually collected and compiled. Additionally, lots of external links were available for more information covering extensive biomedical knowledge. HAMdb provides a user-friendly interface to query, search, browse autophagy modulators and their comprehensive related information. HAMdb will help researchers understand the whole autophagy process and provide detailed information about related diseases. Furthermore, it can give hints for the identification of new diagnostic and therapeutic targets and the discovery of new autophagy modulators. In a word, we hope that HAMdb has the potential to promote the autophagy research in pharmacological and pathophysiological area.

## Background

Autophagy is a highly evolved and highly conservative process in eukaryotic organisms for degradation and recycling of biomolecules and damaged organelles. Commonly, there are three discrete types of autophagy: microautophagy, chaperone-mediated autophagy (CMA), and macroautophagy. For microautophagy, the lysosome itself engulfs small components of the cytoplasm for degradation by inward invagination of the lysosomal membrane [1]. CMA is a specific degradation pathway for cytosolic proteins that contain a KFERQ-like pentapeptide. In this process, chaperone protein Hsc70 (heat shock cognate 70) and cochaperones specifically recognize these special proteins and their complex can be delivered into the lysosomal lumen for degradation through a transmembrane protein Lamp-2A [2]. Macroautophagy, as the major type of autophagy, has been studied most extensively compared to microautophagy and CMA, and thus hereafter referred to as autophagy. It is mainly mediated by autophagy-related proteins and involves an intermediate organelle named autophagosome. Although different types of autophagy occur in different ways, they all play an important role in the process of responding to outside stimulation and removing damaged substances [3–6].

Autophagy consists of several sequential steps: sequestration, transport to lysosomes, degradation, and utilization of degradation products [7, 8]. Recent studies have clearly demonstrated that autophagy has a greater variety of physiological and pathophysiological roles than expected and each step can have a variety of physiological roles. Despite efforts to assign known functions to individual steps, many proposed functions remain to be assigned. Accordingly, autophagy is now widely implicated in pathophysiological processes (*e.g*., cancer, infection diseases, metabolic and neurodegenerative disorders, and cardiovascular and pulmonary diseases) and in physiological responses to exercise and aging [9–12]. Considering the key role of autophagy in cell biology and its considerable therapeutic potential, the discovery of its modulators may be a new strategy for clinical therapy [13]. Recently, a lot of experiments indicate that inhibiting autophagy can increase the sensitivity of tumor cells to radiotherapy, chemotherapy and molecular targeted drugs, increase cell death and thus enhance the treatment efficacy. Among the existing autophagy inhibitors, only anti-malarial drugs chloroquine and hydroxychloroquine are used in clinical trials to increase survival rates for cancer patients because their clear pharmacological and toxicological properties [14–16]. Actually, current therapeutic targeting of autophagy in human disease is still limited although many efforts have been made in this direction mainly due to following several reasons: (1) an incomplete understanding of how the process contributes to pathogenesis; (2) the lack of specificity of compounds that can influence autophagy; (3) the limited availability of candidate therapeutics with clinical efficacy [17–19]. That is, the comprehensive understanding of autophagy-related genes, proteins and modulators (*e.g*., know about their explicit role, target, pathway and involved disease information) will be of great help for identifying new diagnostic and therapeutic targets and developing new autophagy modulators. To our best knowledge, there are already some databases specializing in autophagy available [20, 21]. However, they only involve a part of information of autophagy modulators or only cover biological and structural information of autophagy-related proteins and lack corresponding pharmacological and pathophysiological information. Therefore, the specific databases covering autophagy modulator (proteins, chemicals and microRNA)-related target, pathway and disease information are urgently needed to autophagy research and drug discovery.

To give impetus to further study and promote the autophagy research in pathophysiological area, we try to collect the functional mechanism (specific effect on autophagy), pathway and disease information of existing autophagy-related modulators (proteins, chemicals and microRNAs) as many as possible. Additionally, some basic information (biological, structural and physicochemical information) and most commonly used links are also added to provide more detailed information. A specialized database is built and freely available at http://hamdb.scbdd.com for the publics to make effective utilization of these available information.

## Materials and methods

### Data collection

To provide comprehensive information of autophagy modulators for researchers, we searched not only for related proteins, but also related chemicals and microRNAs from peer-reviewed literatures, available databases and some websites. The detailed collection processes are described as follows.

Related proteins: Firstly, we searched and reviewed autophagy-related scientific articles recorded by PubMed as many as possible and extracted some useful information for us. In this step, we collected 545 autophagy-related genes from 499 literatures after removing duplicates. For these genes, their molecular type, specific effects on autophagy (*e.g.*, their increased/decreased activity will increase/decrease autophagy), species evidence and corresponding experimental references were reserved. Additionally, their pathway and disease information have also been added including canonical pathways, downstream microRNAs, proteins and chemicals, upstream proteins and chemicals, role in cell, involved disease, OMIM information, KEGG disease information. After that, we searched for the autophagy-related database and found two excellent databases: Human Autophagy Database (HADb, http://www.autophagy.lu/) and the autophagy database (http://www.tanpaku.org/autophagy/, human). From them, we obtained 251 new related genes and their pathway information were collected from Autophagy Regulatory Network database. For all the collected genes, their corresponding uniport ID (Homo sapiens) and protein description were compiled manually. And then, 20 external database links containing structural and biological information were added: Gene ID, GI number, Uni Gene, PDB, disport, BioGrid, MINT, String, ChEMBL, DrugBank, Guide to Phar, Swisslipids, Biomuta, Ensembl protein, KEGG, Pharm GKB, Biocyc, Reactome, Unipathway, and Gene wiki.

Related chemicals: Similar to the protein collection process, we firstly collected 246 related chemicals from 367 literatures recorded by PubMed. For these chemicals, their molecular type, specific effects on autophagy (*e.g.*, their increased/decreased activity will increase/decrease autophagy), species evidence and corresponding experimental references were reserved. Additionally, some pathway and disease information including target, pathway, biological description and corresponding gene name listed in aforementioned protein database. After that, we also obtained 595 new chemicals from MedChem Express, Selleck and APExBIO. Their research area, category (activator/inhibitor), in vitro*/vivo* test, clinical trials were reserved. For all the chemicals, some basic information was collected: IUPAC name, alternative names, canonical SMILES, molecular formula, molecular weight, solubility. Furthermore, 18 important physicochemical and ADME properties were calculated by our ADMETlab platform and chemopy package [22]: hydrogen acceptor, hydrogen donor, logD (pH = 7), pKa (pH = 7), pKb (pH = 7), druglike, logP(o/w), logS, SlogP, TPSA, loghERG, Caco-2, logBB, MDCK, logKp, logKhsa, human oral absorption, and percent human oral absorption (%). Four external links including structural and drug information were added: CAS number, PubChem CID, HMDB ID, and DrugBank ID.

Related microRNAs: In this part, we totally collected 132 autophagy related microRNAs from literatures recorded by PubMed and a noncoding RNA database, ncRDeathDB (www.rna-society.org/ncrdeathdb) after removing some duplicates [23]. Their molecular type, specific effects on autophagy (*e.g.*, their increased/decreased activity will increase/decrease autophagy), species evidence and corresponding experimental references were reserved. Additionally, the gene description, RefSeq status, organism, synonyms and miRbase ID were also compiled to supply the biological information.

### Database implementation

The HAMdb database was deployed and runs on an ECS (elastic computation service) server of Aliyun Company. The number of CPU cores and memory are automatically allocated to the running instances on demand, which ensures the elastically stretchable computing capability. We have set a long time supporting plan to maintain the database and update new data.

The server-side components were written in Python programming language. An open source Python framework (Django) was employed to develop the Graphical User Interface (GUI). Considering the balance between data capacity and query efficiency of Django, MySQL was chosen as the storage engine of this database. The client-side components were developed in HTML5 and CSS3, using JavaScript and jQuery to help accomplish some complex interaction processes and result visualization. The AJAX technology and JSON data format were used to realize the asynchronous data callback and rendering. The Nginx + uWSGI architecture was used to enable an efficient data exchange between dynamic data from the server-side and static contents form the client-side. The GUI is well designed and runs well on desktop browsers and mobile devices on the major operating systems including Windows, macOS, Linux, iOS, and Android operating systems using modern browsers like Chrome, Firefox and Safari.

A striking feature of HAMDB is the clear and optimized design of database tables and fields. On the one hand, we created parent tables to store the basic information of chemicals, proteins and microRNAs individually; set child tables to extend the related information of each object from the corresponding parent table; set primary and foreign keys to link parent and child tables. On the other hand, we optimized the field type and the size of each table to ensure a relatively small table size. These measures enable us to update records, extend object information and add tables of new kinds of modulators easily and conveniently, which makes the database a sustainable scientific project.

## Result and discussion

### Content of HAMdb

**Fig. 1:**
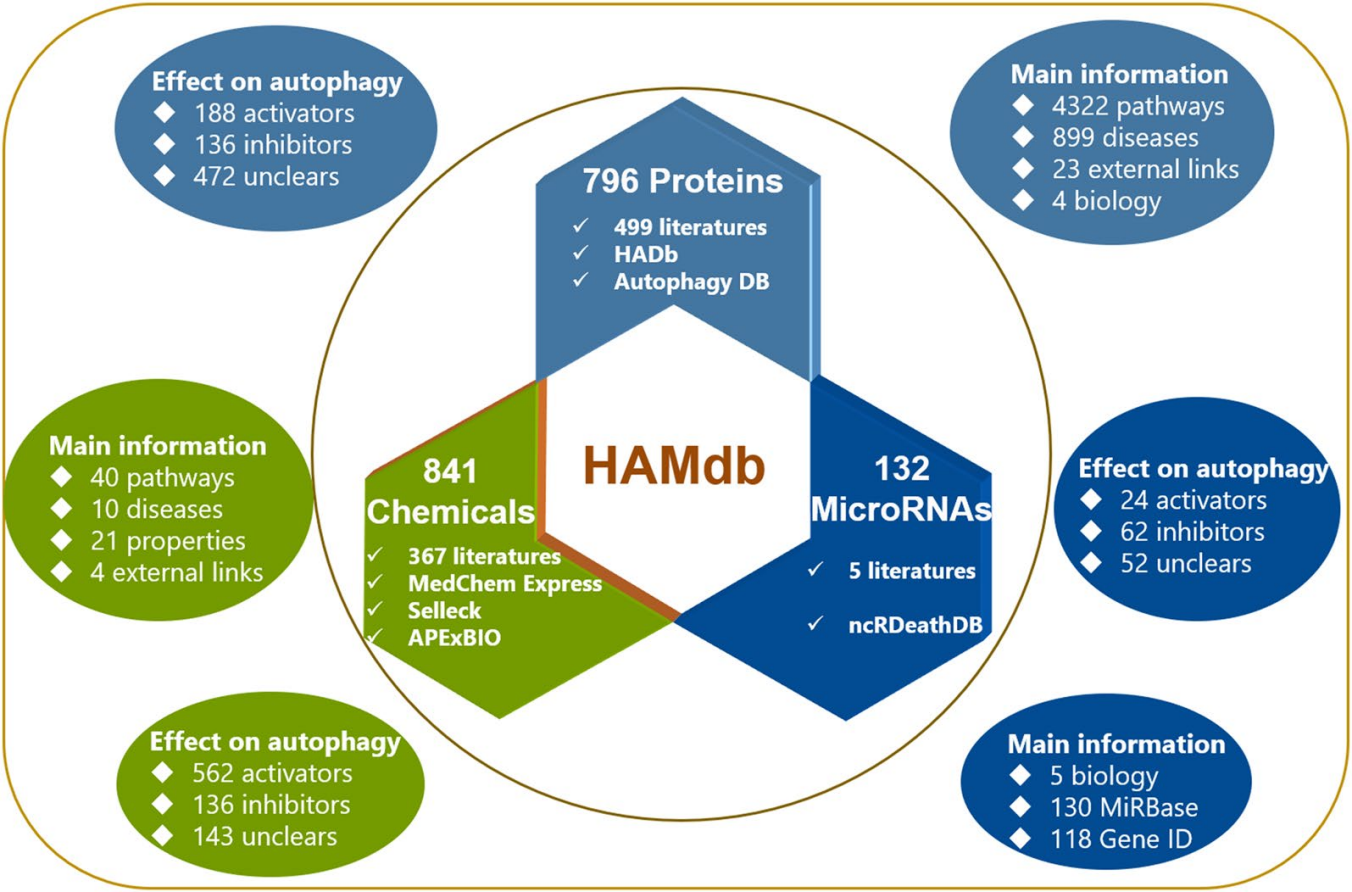
The construction and detailed information of HAMdb. HAMdb contains 796 proteins (collected from 499 literatures, HADb database and Autophagy database), 841 chemicals (collected from 367 literatures, Medchem Express, Selleck and APExBIO) and 132 microRNAs (collected from 5 literatures and ncRDeathDB), their main information and effects on autophagy can be seen in above figure

After the data collection process, we finally obtained 796 proteins, 841 chemicals and 132 microRNAs. And, their basic information, specific effect, pathway and disease information were comprehensively compiled as described before. Detailedly, 796 related proteins involved 501 published literatures, 86 cell lines, 4322 pathways and 899 diseases. Further, we artificially divided their effects on autophagy into three types: activator, inhibitor and unclear. Autophagy activators refer to modulators that have positive effects on autophagy: increased activity increases autophagy (*e.g.*, ABHD5), decreased activity decreases autophagy (*e.g.*, CDKN1B) and chemicals that enhance autophagy levels (*e.g.*, Temozolomide). Similarly, the autophagy inhibitors are the modulators that have negative effect on autophagy: increased activity decreases autophagy (*e.g.*, AGTR2), decreased activity increases autophagy (*e.g.*, AKT2) and chemicals that reduce autophagy levels (*e.g.*, Chloroquine). The unclear group includes proteins that have no, have no clear or have contradictory effect information. For instance, BCL2L1 was recognized as an unclear protein due to that increased BCL2L1 increases autophagy of colorectal cancer cell lines, but for cervical cancer cell lines, it acts as an inhibitor. Accordingly, there were 188 activators, 136 inhibitors and 472 unclears. Similarly, 367 scientific articles, 82 cell lines, 40 pathways and 10 categories of diseases were connected with 841 chemicals. Among them, there were 562 activators, 136 inhibitors and 143 unclears. As to 132 autophagy-related microRNAs, they involved 118 cell lines, 24 of them are activators, 56 microRNAs are inhibitors and the rest 52 are unclears. In the future, we plan to update HAMdb database annually from following aspects: add new autophagy related proteins, chemicals, microRNAs based on peer-reviewed publication; add new pathway and disease information of existing autophagy modulators; add more search function in updated versions. These autophagy modulators are the core content of HAMdb and can give researchers a new understanding of autophagy in views of pathways and human diseases, their detailed information was shown in Fig. 1.

### HAMdb website

**Fig. 2:**
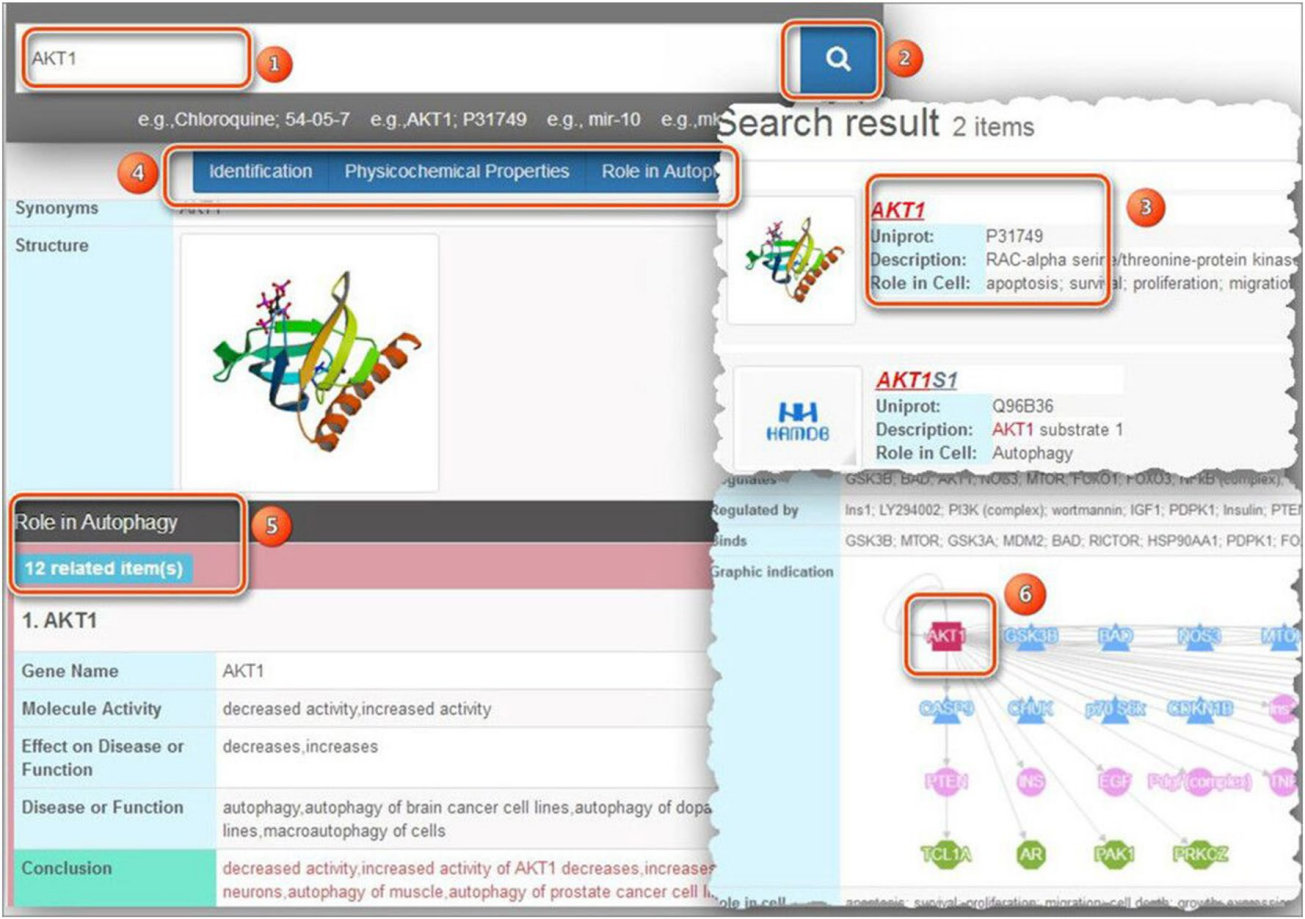
The flowchart for retrieving HAMdb related entry. (1/2). Different ways can be used for searching. (3). The search result of a representative querying entry. (4/5/6). The detailed information of an autophagy-related entry

HAMdb website is available at http://hamdb.scbdd.com. The website is designed to give a comfortable way for data querying, searching, browsing, and visualization. HAMdb consists of four main functional modules: search, browse, download and contact. Users can search a modulator by several different ways: For chemical searching, general name, synonyms, trade names terms and CAS number can be used as an input (Example: search for chloroquine, the following terms can be used: “chloroquine”, “Aralen” or “54-05-7”). A specific protein can query using gene name, synonyms, and uniport ID (Example: search for AKT1 can query using “AKT1” or “P31749”). As to microRNA, the general name can be used for searching (Example: mir-10 can be queried using “mir-10”). And then, the result page will list related items and their basic information. With click on an item, more detailed descriptions such as identification, physicochemical properties, role in autophagy, external links and the references are displayed in the “Detail” page. In order to give an insightful view of the biological behaviors of the query item, the page renders a graphic picture to demonstrate the relationship of the “Regulates”, “Regulated by” and “Binds” by using lines and shapes in different colors. The flowchart for retrieving can be seen in Fig. 2. The browse module enables users to browse the autophagy modulators in alphabetical order and different cell lines. As to the download module, users can download all the data in “Microsoft Excel”, “CSV” or “SDF” format on their own. If users have any questions or comments related to the database or other suggestions about our website please contact us as described in the contact module.

### Application

Considering the research states and present challenges of autophagy, we think that HAMdb can be used in following aspects: (1) Help to understand the process. As described before, for each modulator, not only its specific effect and canonical pathway, but also its upstream and downstream are collected to give an overall understanding of its role in the autophagy process. Taking the apoptosis regulator Bcl-2 as an example, from the search result of HAMdb, we can know that increased Bcl-2 activity decreases autophagy of cell/breast cancer cell lines and it may involve PI3 K/AKT/mTOR signaling pathway, STAT3 signaling pathway and TGF-β signaling pathway. Additionally, its upstream elements such as CYCS, CASP3, BAX and downstream elements such as TNF, beta-estradiol, curcumin, TP53 can be detected. Based on these information, we can speculate that Bcl-2 may affect autophagy by regulating PI3 K/AKT/mTOR signaling pathway and consequently can affect the development of breast cancer. In fact, the nucleation phase of autophagy is controlled by a complex involving PI3KCIII with either Beclin1-Atg14L-PI3KCIII-p150, Ambra1 or Beclin1-UVRAG-PI3KCIII-p150-Bif1 and is negatively regulated by the antiapoptotic protein Bcl-2. The over-expression of Bcl-2 can promote the development of breast cancer cells by binding to Beclin 1 and inhibiting autophagy [3]. Clearly, by means of HAMdb, the researchers could conveniently understand the pathway information of autophagy-related proteins. (2) Give detailed information about related disease. In this database, we creatively collect the related disease information for every modulator. Based on these information, users can connect disease and proteins, chemicals or microRNAs, and further study about detailed pathological mechanism may be facilitated. For mTOR, a lot of related diseases such as metastatic breast cancer, non-squamous non-small cell lung cancer, epithelial ovarian cancer, breast cancer are reported. We think that the inhibitor of mTOR may play an important role in the cancer therapeutic field and massive efforts should be made in relevant studies. Up to now, there are already several inhibitors available for mTOR: Sirolimus, a registered anti-rejection drug in kidney transplant recipients [24]; Temsirolimus and Everolimus, registered for renal cancer [25, 26]; NV-128111, under preclinical research [27]. (3) Give hints for new modulators and potential drug candidates. Based on the pathway and upstream/downstream information, a series of new modulators (*e.g*., specific autophagy activators or inhibitors) may be designed and synthesized according to some indirect mechanisms. As an example, we can obtain the upstream elements information of VEGFA from HAMdb, such as KIT, MET, NGFR, PDGFRA, PDGFRB, TEK and so on. Therefore, not only direct autophagy modulators aiming at VEGFA can be designed, but also some indirect modulators acting on KIT, MET or PDGFR deserve much attention. So far as we know, there are at least two related drugs targeting at KIT and PDGFR: Imatinib registered for gastrointestinal stromal tumor and Sorafenib registered for renal cancer and hepatocellular carcinoma [28–30]. (4) Identify new diagnostic and therapeutic targets. By integrating the disease information and the connection of disease and protein/chemical/microRNA, some promising diagnostic and therapeutic targets may be put forward and bring good clinical benefit. For Bcl-2, both of its upstream and downstream elements can be regarded as new therapeutic targets and their changes under pathological state may provide a potential diagnostic means for clinical researchers. For example, not only the expression level of Bcl-2, but also the level of Beclin 1 can be taken as a promising index for breast cancer [31]. (5) Promote the autophagy research in pathophysiological area. As mentioned in the first four items, thanks to comprehensive pathway and disease information included in HAMdb, it will enable researchers to know more about the role of autophagy in different diseases and may facilitate the autophagy study in pharmacological and pathophysiological area.

### Comparison with other resources

In recent years, a lot of efforts have been done to collect autophagy related information and there are already some practical autophagy resources to our best knowledge: Human Autophagy Database (HADb, http://www.autophagy.lu/), the autophagy database (http://www.tanpaku.org/autophagy/), ncRDeathDB (http://www.rna-society.org/ncrdeathdb), Autophagy Regulatory Network (ARN, http://autophagy-regulation.org) [32], autophagic compound database (ACDB, http://www.acdbliulab.com/) [33] and the Autophagy, Necrosis, ApopTosis OrchestratorS (THANATOS, http://thanatos.biocuckoo.org) [34]. Compared with these databases, HAMdb has some unique features that mainly reflects on basic information and functional information. The details of seven autophagy resources were listed in Table 1. From this table, we can see that HADb, AutophagyDB and THANATOS only involve in related proteins and their structural and biological information. But for HAMdb, other modulators such as chemicals and microRNAs are also collected. In fact, HADb mainly describes the features and sequences of the autophagy genes, transcripts, exons and proteins, it only contains 234 autophagy related proteins and their basic structural information. The AutophagyDB aims to integrate a list of autophagy-related proteins and their potential orthologs in 41 eukaryotes and the THANATOS mainly contains proteins potentially associated with autophagy cell death pathways in 164 eukaryotes. Similarly, ncRDeathDB and ACDB also only cover a part of information for autophagy modulators. As to ncRDeathDB, it mainly provides noncoding RNA-associated cell death interactions and helps to visualize and navigate current knowledge of the noncoding RNA component of cell death and autophagy. For this database, it only contains 121 autophagy related microRNAs. And, ACDB only contains information of 357 compounds with 164 corresponding signaling pathways and potential targets in different diseases. In 2015, Tamas Korcsmaros et al. developed a systems-level bioinformatics resource for studying the mechanism and regulation of autophagy named ARN. It not only provides data on post-translational, transcriptional and post-transcriptional regulators, but also makes a connection between the cellular signaling network and the regulation of autophagy. It focuses on the network analysis and helps the investigation of transcription factors, miRNAs and signaling pathways. Compared with ARN, HAMdb pays more attention to specific mechanism of autophagy modulators and related experimental information and study status. It covers not only structural and biological information, but also some functional information including specific effect on autophagy, pathway, disease, upstream, downstream and their corresponding reference. Based on the information obtained from HAMdb, researchers can have an overall understanding of autophagy process and the related pathways. More important, it will help to uncover the relationship between autophagy and various diseases and thus promote the autophagy study in pharmacological and pathophysiological area.

**Table 1:** The details and comparation of 7 autophagy related resources

Database	Basic information				Functional information						
	Protein	Chemical	MiRNA	Structure	Biological	Specific effect	Pathway	Disease	Upstream	Downstream	Reference
HADb	234			✔	✔						
AutophagyDB	582			✔	✔						
ncRDeathDB			121	✔	✔						
ARN	1485*		386*	✔	✔		✔				✔
ACDB		357		✔				✔			✔
THANATOS	4237*			✔	✔		✔				✔
HAMdb	796	841	132	✔	✔	✔	✔	✔	✔	✔	✔

*Not only include human related genes, but also their potential orthologs. Table 1 lists 7 practical autophagy resources and their basic information and functional information, compared with other databases, HAMdb has some unique features and shows its important role in pharmacological and pathophysiological area

## Conclusion

Autophagy, as an essential, conserved lysosomal degradation pathway that controls the quality of the cytoplasm by eliminating protein aggregates and damaged organelles, has been extensively studied in recent years. Not only its basic cellular mechanism, but also its role in human health and disease has become widespread. Considering the key role of autophagy in cell biology and its considerable therapeutic potential for various diseases, the comprehensive understanding of autophagy-related modulators and corresponding pathway and diseases information will be of great help for identifying new diagnostic and therapeutic targets. Inspired by the lack of autophagy-related pathway and disease information, we manually collected the literatures and integrated external resources to gain a high coverage autophagy database. Based on them, we developed an online resource, Human Autophagy Modulator Database (HAMdb, http://hamdb.scbdd.com.), to provide researchers pathway and disease information as many as possible. HAMdb contains 796 proteins, 841 chemicals and 132 microRNAs from 871 scientific literatures and 286 cell lines. Their specific effect on autophagy, physicochemical information, biological information and disease information were carefully collected and compiled. Additionally, a lot of external links were available for more information including sequence database, 3D structure database, protein–protein interaction database, chemistry database and so on. The user-friendly website of HAMdb allows researchers without computational background to query, search and browse the database. The database can be downloaded in Excel, CSV, and SDF file formats. HAMdb will help researchers to understand the whole autophagy process and give detailed information about related diseases. Furthermore, it can give hints for discovery of new modulators and identify new diagnostic and therapeutic targets. In the long run, HAMdb has the potential to promote the autophagy research in pharmacological and pathophysiological area.

## Availability and requirements

- Project name: *HAMdb.*
- Project home page: http://hamdb.scbdd.com.
- Operating system(s): Platform independent.
- Programming language: Python, JavaScript, HTML, CSS.
- Other requirements: Modern internet browser supporting HTML5 and JavaScript. The recommended browsers: Safari, Firefox, Chrome, IE (Ver. >8).
- License: http://creativecommons.org/licenses/by-nc-sa/4.0/.
- Any restrictions to use by non-academics: License needed.
